# Efficacy and safety of 0.05% micellar nano-particulate (MNP) cyclosporine ophthalmic emulsion in the treatment of moderate-to-severe *keratoconjunctivitis sicca*: a 12-week, multicenter, randomized, active-controlled trial

**DOI:** 10.1186/s12886-023-02838-z

**Published:** 2023-03-27

**Authors:** A Tarakeswara Rao, Amit Gupta, Tulika Chauhan, Sayan Basu, Nitin Batra, Namrata Sharma, Virender S Sangwan, Vinay Gupta, Shoibal Mukherjee

**Affiliations:** 1 Rajiv Gandhi Institute of Medical Sciences and RIMS Government Hospital, Srikakulam, 532001 India; 2 Post Graduate Institute of Medical Education and Research, Chandigarh, 120012 India; 3 Centre for Sight Eye Institute, New Delhi, 110075 India; 4 LV Prasad Eye Institute, Hyderabad, 500034 India; 5 Christian Medical College & Hospital, Ludhiana, 141008 India; 6 All India Institute of Medical Sciences, New Delhi, 110029 India; 7 Dr Shroff’s Charity Eye Hospital, New Delhi, 110002 India; 8 JSS Medical Research Asia Pacific, Faridabad, 121003 India; 9 New India Biopharma, New Delhi, 110065 India

**Keywords:** Cyclosporine, Keratoconjunctivitis sicca, Dry eye disease, Micellar nanoparticle, Corneal fluorescein staining

## Abstract

**Background:**

Keratoconjunctivitis sicca or dry eye disease (DED) is a multifactorial disorder underpinned by a complex inflammatory cycle. Introduction of topical cyclosporine has been a significant advance in the management of DED. In recent years advancements in formulation technology have led to development of micellar nano-particulate (MNP) cyclosporine formulations that promise better penetration into ocular target tissues and potential for reduced ocular surface irritation.

**Methods:**

We compared two dosing regimes of a proprietary MNP cyclosporine emulsion with the widely marketed topical cyclosporine formulation Restasis™ in a multicenter parallel-group randomised trial in patients with DED. Patients were randomised to one of 3 treatment groups with 90 patients eligible for the per protocol analysis: 30 in the higher dose test arm A; 32 in the lower dose test arm B; and 28 in the Restasis™ control arm C. All scored efficacy endpoints were tested for significance by comparing the mean change in scores from baseline in the test groups with that in the control group at 12 weeks, using the Student’s t test. Wilcoxon’s rank sum test was used to test individual symptom scores and clinician’s global evaluation of treatment grades.

**Results:**

Corneal fluorescein staining score, the primary efficacy endpoint, decreased by 6.8 ± 4.0, 5.7 ± 3.9, and 4.6 ± 3.6 points in the 3 groups respectively, indicating superior efficacy in test arm A in comparison to control arm C (p = 0.0026). Schirmer’s tear test, conjunctival lissamine staining score, ocular surface disease index, and individual dry eye symptom scores also favoured higher dose MNP cyclosporine over Restasis™. The study failed to differentiate the treatment arms in terms of clinician’s global evaluation of treatment, use of tear substitutes, best corrected visual acuity or safety and toleration.

**Conclusion:**

The results indicate that the dose of 1 drop of a 0.05% w/v ophthalmic emulsion of MNP cyclosporine administered topically twice daily yields better outcomes at 12 weeks than the lower dose tested in the study, and is more efficacious than an equivalent dose of Restasis™, the active control used in the study.

**Trial registration:**

This trial was registered in the Clinical Trials Registry of India on 29/03/2019, and was assigned registration number CTRI/2019/03/018319.

## Background

Dry eye disease (DED) is the most prevalent ocular surface disorder with prevalence of over 10% in individuals ≥ 50 years of age [1]. A chronic inflammatory cycle involving numerous cytokines, cells and mediators, underlies the pathophysiology of DED [2]. Cyclosporine, first introduced for the prevention of organ transplant rejection, has been demonstrated to possess a wide spectrum of anti-inflammatory, immunosuppressive, and immunomodulatory actions on the ocular surface [3]. Following approval of a topical ophthalmic formulation of the drug by the US FDA in 2003, cyclosporine A (CsA) has secured an established place in the treatment of DED. The conventionally formulated oil-in-water emulation of the drug has, however, not shown consistent results in clinical trials [4]. Moreover, while statistically significant improvements in signs and symptoms of disease have been documented, the effect size has been small, with the Restasis™ prescribing information stating that an increase to the clinically meaningful value of over 10 mm/5min Schirmer wetting was seen in approximately 15% of patients versus approximately 5% of vehicle-treated patients [5]. Additionally, there is scope for improvement in toleration, with over 20% of patients who received Restasis™ in pivotal trials reporting burning, stinging or other discomforts in the eyes. CsA delivered via conventional oil-based topical formulations has limited penetrability into ocular tissues [6, 7]. It is therefore uncertain whether the bioavailability of CsA from conventional topical ophthalmic formulations in the various ocular target tissues involved in the pathophysiology of DED is optimal for best clinical results. Micellar Nanoparticle (MNP) Cyclosporine is a novel topical ophthalmic formulation of CsA that delivers the active ingredient to the ocular surface in neutral to positively changed (mean ζ potential range−5 to + 55 mV) nano-sized particles (mean particle size range 150 to 170 nm) in aqueous phase within amphiphilic micelles in addition to dissolved CsA in nano-sized oil globules. The surface charge range and biphasic presentation of CsA in the formulation is expected to increase the availability of CsA for action at deeper layers of the cornea and other tissues of the ocular surface, thereby improving efficacy without adversely affecting toleration [8]. The present study was planned to test this hypothesis.

## Methods

We undertook a 24 week, randomized, multicenter, open-label, parallel group study in 155 adult patients with moderate to severe keratoconjunctivitis sicca to evaluate the efficacy and safety of a proprietary formulation of MNP Cyclosporine (0.05% w/v, New India Biopharma Pvt. Ltd.) applied to the ocular surface either once or twice a day as compared to RESTASIS™ (cyclosporine 0.05% w/v, Allergan India Pvt. Ltd.) applied to the ocular surface twice a day. An in vitro study using the Franz diffusion cells apparatus showed 3-fold higher retention in corneal tissue and significantly higher transcorneal release of CsA with MNP Cyclosporine than with RESTASIS™ (unpublished data on file), suggesting the possibility of superior clinical efficacy of MNP Cyclosporine over RESTASIS™. However, previous studies with RESTASIS™ had reported a lack of incremental dose response at concentrations above 0.05%^9^. Thus the 0.05% strength, at the same dose as recommended for RESTASIS™, was chosen for primary comparison. A lower dose arm of MNP Cyclosporine was added to cover the possibility of toleration issues in relation to higher corneal retention and transcorneal release of CsA. Objective and subjective measures of ocular surface disease activity were assessed at baseline and at the end of 4, 8, 12 and 24 weeks. Safety assessments were performed throughout the study. As turnout for the fourth and final follow-up visit at the end of 24 weeks of treatment was severely disrupted due to pandemic associated movement restrictions, we present here the results observed up to the end of 12 weeks of treatment with study drugs by evaluation of tear production, ocular surface integrity and subjective symptoms. The study was carried out at 7 clinical sites. An institutional ethics committee (IEC) reviewed and approved the protocol at each site. Written informed consent was obtained from each patient prior to study participation. The study was conducted in accordance with the principles of Good Clinical Practice and applicable regulations pertaining to clinical trials.

### Study design

Patients with at least 6 months’ history of keratoconjunctivitis sicca (KCS), who had not used cyclosporine in any form in the previous 90 days, and complained of an inadequate response to ongoing treatment were screened for eligibility to participate in the study. Eligible patients were asked to discontinue all previous treatment for DED other than tear substitutes, and were randomly assigned to one of three treatment arms in 1:1:1 ratio on the basis of a randomization schedule maintained at a central location off-site. The treatment arms were as follows:

Treatment Arm A – MNP Cyclosporine 0.05% twice daily for the entire duration of the trial.

Treatment Arm B – MNP Cyclosporine 0.05% twice daily for the first 4 weeks, and once daily for the remaining duration of the trial.

Treatment Arm C – Restasis™ 0.05% twice daily for the entire duration of the trial.

Patients were instructed to instil a single drop of the drug at each administration – once in the morning on waking and again at bedtime. Patients in Arm B were advised to administer the drug only at bedtime during the last 20 weeks of treatment. The use of tear substitutes was permitted and patients were provided with packs of Refresh Tears™ to be instilled into the eyes as required, but no more than 8 times a day, separated from study drug administration by at least 30 min. Study participants were asked to keep a calendar record of drug administration, symptoms of ocular side effects following drug administration, and the use of tear substitutes. Follow-up visits were scheduled 4, 8, 12 and 24 weeks after the start of treatment.

### Study population

Patients ≥ 18 and ≤ 65 years of age reporting to the outpatients clinics of participating sites with moderate to severe KCS as evidenced by the following measures of disease activity were eligible for the study: (1) Schirmer Tear Test (STT) value (without anaesthesia) ≤ 10 mm/5 min in at least one eye; (2) Corneal fluorescein staining (CFS) score AND/OR conjunctival lissamine green staining (CLGS) score of ≥ 4 in either eye using the National Eye Institute (NEI) scale; (3) At least one ocular symptom assessed to be at moderate severity or worse from among the following: photophobia, blurred vision, foreign body sensation, soreness or pain, itching, burning, and dryness; (4) Ocular Surface Disease Index (OSDI) ≥ 20. Patients were required to have best corrected visual acuity (BCVA) of 0.7 log MAR or better in each eye as assessed using an ETDRS chart, and normal eyelid position and closure.

Exclusion criteria required patients to be free of other ocular and systemic ailments (including Sjögren’s syndrome), as well as concomitant medication, that could interfere with the study or the interpretation of its efficacy or safety results. Patients with recent eye surgery, those with punctal plugs or needing contact lenses, and those who had failed previous treatment with cyclosporine were also excluded.

### Randomisation

A sequence for random allocation of patients to the 3 treatment groups was generated by an isolated biostatistician within the organization responsible for study management. The PLAN procedure provided in the statistical software package SAS version 9.4 was used with block size of 6. Prospective concealment of the sequence was achieved using the envelope method. Patients were enrolled separately at each study site, and interventions were assigned to patients by an investigator at each site on the basis of the assignment revealed on opening envelopes in sequence at the time of patient enrolment.

### Efficacy assessments

Mean change from baseline in CFS scores at 12 weeks was taken as the primary efficacy endpoint, while STT value, CLGS score, OSDI, individual dry eye symptom scores, clinician’s global evaluation of treatment (CGET), and use of tear substitutes, served as secondary efficacy endpoints.

*CFS scores* were obtained by grading the intensity of staining observed 1 min after ocular administration of fluorescein dye. The NEI / Industry Workshop classification system was used [10]. Employing a yellow barrier filter, the slit lamp’s cobalt blue illumination, and a grading scale of 0 to 3, the sum of grades assigned to each of 5 zones of the cornea was taken as the CFS score. This system provides a score range of 0 to 15.

*STT* was performed without anaesthesia. The subjects were seated with their eyes closed and the lower cul-de-sac was gently dried with a cotton applicator. Sterile Schirmer Strips were inserted into the lower conjunctival sac at the junction of the lateral and middle thirds, avoiding touching the cornea. After five minutes, during which the subject were instructed to keep the eyes open and to blink normally, the strip was removed and measured to the point of maximum wetting. The amount of wetting measured in millimetres (mm) using a graduated paper scale was taken as the STT value.

*Conjunctival staining* was graded using the NEI / Industry Workshop classification system [10]. The conjunctiva was graded on a scale of 0 to 3 according to the intensity of lissamine green staining in three zones each of the nasal and temporal bulbar conjunctiva. CLGS score was computed as a summation of grades assigned to each zone. This system provides a score range of 0 to 18.

*OSDI* was calculated based on patient responses to the 12-item questionnaire and the number of questions scored – questions not applicable or not answered being excluded. Each question was scored from 0 to 4 on the basis of frequency of symptoms expressed by the patient. The sum of scores assigned to each question was divided by the number of questions scored, and the quotient multiplied by 25 to get the OSDI.

At baseline and each follow-up visit, patients were provided with a questionnaire and asked to score seven individual symptoms of dry eye disease (*dry eye symptom scores* – *DESS*) on a 5-point scale from 0 (absent) to 4 (always) on the basis of frequency of occurrence immediately preceding the visit. The symptoms listed in the questionnaire were: blurring of vision; feeling of dryness of eyes; itchy eyes; sensitivity to light; painful or sore eyes; feeling of sandiness or grittiness in eyes; and feeling of stinging or burning eyes.

Investigators were required to grade each patient for treatment effectiveness at each follow-up visit. The *CGET grading* uses a 7-point ordinal scale with worsening of the patient’s condition at the lowest end of the scale and complete clearing of all signs and symptoms at the highest end.

Evaluation of the use of tear substitutes was based on count of dispensed packs returned at each follow-up visit.

### Safety assessment

Safety was assessed on the basis of incidence and severity of adverse events noted by investigators or reported by study participants at any time during the course of the study. Adverse events, whether local to the eye or systemic, were defined as any unfavourable or untoward sign, symptom or disease that occurred during the course of the trial irrespective of a causal relationship to study drugs. Adverse events would be classified as “serious” if they met criteria for seriousness defined in the International Conference on Harmonization (ICH) E2A guideline. Investigators were required to state their opinion on causal relationship of adverse events to the investigational products based on the WHO-UMC system for standardised case causality assessment.

### Statistical analysis

At 80% power and 2.5% one-sided level of significance, an estimated sample size of 79 would be expected to detect a non-inferiority margin of 0.2 for the mean difference in the primary endpoint with standard deviation of 0.45. Considering a drop out rate of 20%, the final sample size for each arm was set at 100 eyes (50 patients). Datasets generated through compilation of data entered into an electronic data capture system (Clinion v3.0) by investigators and/or authorized site staff were analysed using standard statistical analysis software (SAS v9.4). Individual patient data was included or excluded from analysis based on criteria defined for the ‘intention-to-treat’ (ITT) dataset and the ‘per-protocol’ (PP) dataset. The ITT dataset included all randomized subjects who were available for at least one post-baseline assessment. In case of missing data at a scheduled assessment time point in the ITT dataset, the last available observation was carried forward. The PP population was defined as all subjects who were available for the specified assessment within the time window permitted by the study protocol without any protocol deviations that may affect the efficacy of the drug, subject safety, or subject’s rights, including, but not limited to, violation of selection criteria, violation of study or assessment procedures, use of prohibited medications, and inadequate (< 75%) study drug compliance as reported by the patient or assessed by the investigator. All patients who received at least one dose of study medication were included in the safety analysis.

All scored efficacy endpoints were tested for significance by comparing the mean change in scores from baseline in the test groups with that in the control group, using the Student’s t test with α value of 0.05. For each patient, both eyes were individually included in the analysis. Wilcoxon’s rank sum test was used to test individual symptom scores and CGET grades.

Adverse events (AEs) were coded by system organ class (SOC) and preferred term using the Medical Dictionary for Drug Regulatory Affairs (MedDRA) version 20.1, and tabulated by treatment group, to indicate the number of patients experiencing each event and number of events experienced. AEs were also evaluated and tabulated by relation to study drug, seriousness, severity (using US NCI-CTCAE v3.0 criteria), and outcome.

## Results

The study enrolled patients from May 2019 to March 2020. A total of 155 patients were randomised to one of 3 treatment groups: 51 to test arm A; 53 to test arm B; and 51 to control arm C. All randomised patients received at least 1 dose of the assigned study drug and therefore the safety population comprised of 155 patients. However, 10 of these patients either withdrew consent or were not available for at least one post-baseline assessment. Thus the ITT population consisted of 145 patients. Of these, 90 patients were able to meet the commitment for a physical follow-up visit within the permitted visit window at the end of 12 weeks of treatment (despite pandemic-related restrictions) and fulfil other eligibility criteria for the PP analysis. Baseline demographics is shown in Table 1.

**Table 1:** Demographics of Analysed Study Populations

Treatment group	A	B	C
Safety population (N)	51	53	51
Sex (count): M/F (M%)	25/26 (49.0%)	27/26 (50.9%)	24/27 (47.1%)
Age (yrs):Mean (SD)	43.4 (11.9)	40.7 (15.1)	45.5 (13.7)
Range	21–69	19–77	19–67
ITT population (N)	48	49	48
Sex (count): M/F (M%)	24/24 (50.0%)	25/24 (51.0%)	23/25 (47.9%)
Age (yrs):Mean (SD)	43.4 (12.1)	40.7 (15.3)	46.3 (13.5)
Range	21–65	19–77	19–67
PP population (N)	30	32	28
Sex (count): M/F (M%)	15/15 (50.0%)	16/16 (50.0%)	12/16 (42.9%)
Age (yrs):Mean (SD)	43.6 (11.4)	40.0 (13.9)	44.6 (13.8)
Range	21–65	19–64	19–65

Of the 55 patients who had to be excluded from the PP population, 44 were not able to honour the trial visit schedule due to pandemic-related restrictions and were unavailable for the crucial 3^rd^ follow-up visit. These included 16 patients who had been randomized to test group A, 15 to test group B, and 13 to control group C. Of the remaining 11 patients further excluded from the PP population, 6 had to be dropped from analysis as pandemic-related restrictions precluded timely supply of study medication to these patients. Three patients were excluded for breach of inclusion criteria, and 2 patients were excluded because they received concomitant medication that was prohibited by the protocol.

Treatment groups were apparently comparable for demographics, pre-existing illnesses, and concomitant medication at baseline other than 4 and 2 patients with a history of thyroid deficiency and related medication in test groups A and B against none in the control group. Further, there were no apparent differences in baseline scores for any of the endpoints assessed for the study. No statistical tests were performed to assess baseline comparability of treatment groups [11, 12].

### Corneal fluorescein staining

The mean CFS score dropped significantly in all groups by the end of 4 weeks of treatment with cyclosporine, and continued to decline thereafter. Scores for the ITT and PP populations at baseline and on follow-up at 12 weeks is shown in Table 2. The reduction in CFS scores was numerically greater in the test groups in comparison to controls, and reached statistical significance for the PP population. While reduction of CFS scores was numerically greater in treatment group A (higher dose) than in treatment group B (lower dose), the difference did not reach statistical significance. Analysis of covariance for difference in mean change from baseline vs. control, with baseline value as covariate, showed statistical significance for treatment group A in both ITT and PP populations but not for treatment group B in either population.

**Table 2:** Effect on Ocular Surface Disease Activity

	ITT population			PP population		
Treatment group	A	B	C	A	B	C
Number of eyes	96	98	96	60	64	56
**Restoration of corneal surface integrity: Corneal Fluorescein Staining Scores (CFSS)**						
Mean score at baseline (± SD)	8.7 (3.4)	9.5 (3.5)	8.7 (3.6)	8.9 (3.0)	9.5 (3.3)	8.5 (3.1)
Mean score at 12 weeks (± SD)	3.3 (3.4)	3.9 (3.0)	4.5 (2.9)	2.2 (2.2)	3.8 (3.4)	3.9 (3.2)
Mean change from baseline (± SD)	− 5.3 (4.1)	− 5.6 (3.8)	− 4.4 (3.7)	− 6.8 (4.0)	− 5.7 (3.9)	− 4.6 (3.6)
P value^†^ (test groups vs. control)	0.0912	0.0247	-	0.0026	0.1101	-
P value^†^ (A vs. B)	0.6475	-	-	0.1329	-	-
ANCOVA^‡^ P value	0.0142	0.0641	-	0.0007	0.4101	-
**Effect on tear formation: Shirmer Tear Test Values (STT)**						
Mean score at baseline (± SD)	6.4 (3.0)	6.7 (3.2)	6.3 (3.6)	6.8 (2.9)	6.6 (3.1)	5.9 (4.0)
Mean score at 12 weeks (± SD)	17.5 (10.5)	15.9 (9.4)	15.1 (8.6)	21.0 (10.3)	17.3 (9.7)	15.8 (9.9)
Mean change from baseline (± SD)	11.1 (9.9)	9.2 (8.4)	8.8 (7.1)	14.2 (9.9)	10.7 (8.3)	10.0 (7.8)
P value^†^ (test groups vs. control)	0.0634	0.7115	-	0.0129	0.6483	-
P value^†^ (A vs. B)	0.1457	-	-	0.0334	-	-
ANCOVA^‡^ P value	0.0610	0.7302	-	0.0120	0.7064	-
**Healing of conjunctival lesions: Conjunctival Lissamine Green Staining (CLGS)**						
Mean score at baseline (± SD)	9.4 (4.6)	10.2 (4.4)	9.7 (4.7)	10.3 (4.3)	10.5 (4.1)	9.5 (3.7)
Mean score at 12 weeks (± SD)	4.1 (3.6)	4.6 (3.3)	5.0 (4.0)	3.1 (3.0)	4.3 (3.3)	4.8 (4.1)
Mean change from baseline (± SD)	− 5.3 (5.5)	− 5.5 (4.8)	− 4.8 (5.0)	− 7.2 (5.9)	− 6.2 (4.7)	− 4.7 (5.3)
P value^†^ (test groups vs. control)	0.4924	0.2736	-	0.0203	0.1066	-
P value^†^ (A vs. B)	0.7375	-	-	0.3251	-	-
ANCOVA^‡^ P value	0.1094	0.3066	-	0.0065	0.3069	-
**Patient-reported improvement in disease severity: Ocular Surface Disease Index (OSDI)**						
Number of patients	48	49	47	30	32	28
Mean score at baseline (± SD)	54.4 (23.4)	55.0 (23.2)	52.5 (26.1)	56.8 (23.5)	56.9 (23.6)	45.9 (21.7)
Mean score at 12 weeks (± SD)	22.6 (18.1)	24.1 (16.3)	25.2 (12.2)	17.3 (15.6)	23.6 (16.8)	22.3 (11.0)
Mean change from baseline (± SD)	− 31.8 (27.2)	− 30.9 (24.2)	− 28.0 (25.5)	− 39.5 (28.7)	− 33.3 (22.1)	− 23.6 (21.8)
P value^†^ (test groups vs. control)	0.4875	0.5757	-	0.0217	0.0911	-
P value^†^ (A vs. B)	0.8605	-	-	0.3464	-	-
ANCOVA^‡^ P value	0.3644	0.6249	-	0.0276	0.4583	-

† Independent samples Student’s t test

‡ Difference in mean change from baseline vs. control, with baseline value as covariate

### Schirmer tear test

Wetting was well below 10 mm at baseline but was more than 10 mm for all groups after 4 weeks of treatment with cyclosporine. Values for the ITT and PP populations at baseline and on follow-up at 12 weeks is shown in Table 2. The increase in wetting of test strips at 12 weeks was evident for treatment group A (higher dose), reaching statistical significance in the PP population. Treatment group B (lower dose) did not differentiate well from Restasis™ in either analysis, and the difference in efficacy of the two dosing regimes showed statistical superiority for the higher dose in the PP population. Analysis of covariance for difference in mean change from baseline vs. control, with baseline value as covariate, showed statistical significance for treatment group A in the PP population but fell short of statistical significance in the ITT population, and treatment group B did not show differentiation from the control group in either population.

### Conjunctival lissamine green staining

Baseline scores averaged around 10 points on the 18-point scale, and reduced significantly through the treatment period in all treatment groups. Table 2 provides the mean scores observed at baseline and at the end of 12 weeks of treatment. The pattern of scores was similar to that observed for corneal staining. Treatment group A showed statistically superior healing efficacy at 12 weeks in comparison to the active control while the difference in scores versus control group did not reach statistical significance for treatment group B. The difference between groups A and B was also not significant. Analysis of covariance with baseline value as covariate confirmed statistical superiority of the higher dose of MNP cyclosporine over the control group in the PP population.

### Ocular surface disease index

The OSDI correlates well with other measures of disease severity in dry eye disease and has been found suitable as a surrogate measure of disease severity in clinical trials. Changes in scores on this 100-point scale based on patient responses to the 12-item questionnaire were in line with other assessments in this study. The higher dose of MNP Cyclosporine was statistically superior to Restasis™ after 12 weeks of treatment with either product in per-protocol analysis. While results with the lower dose of the test product did not reach statistical significance in comparison to the control group, results of the higher and lower dose groups of test product could also not be statistically separated. Analysis of covariance with baseline value as covariate confirmed statistical superiority of the higher dose of MNP cyclosporine over the control group in the PP population. OSDI values at baseline and after 12 weeks of treatment are shown in Table 2.

### Individual dry eye symptom scores

Effect of treatment on individual symptoms of dry eye disease (DESS) was sought to the evaluated through patient-reported scores on 7 discrete symptoms of DED: blurring of vision; feeling of dryness of eyes; itchy eyes; sensitivity to light; painful or sore eyes; feeling of sandiness or grittiness in eyes; and feeling of stinging or burning eyes. Improvement in frequency of all symptoms was evident in all treatment groups for all symptoms from the very first follow-up evaluation in both ITT and PP populations. The differentiation between treatment groups was relatively weak in the ITT population, although a consistent superiority of improvement scores in group A over group C was visible for feeling of dryness of eyes. The scores showed a much stronger and more consistent signal across symptoms and across evaluation time-points in the PP population, where test group A was superior to the control group at the end of 12 weeks for all the 7 symptoms evaluated. The signal was most consistent across evaluation time-points for blurring of vision, feeling of dryness of eyes, photosensitivity, sandy or gritty eyes and stinging or burning sensation in eyes. Superiority of group B over group C was also evident for some of these symptoms. The higher dose of MNP Cyclosporine was significantly more effective than the lower dose in reducing 4 of the 7 evaluated symptoms. Table 3 shows scores and p values for the PP population at baseline and end of 12 weeks.

**Table 3:** Patient-reported improvement in individual symptoms (PP population; N: A = 60, B = 64, C = 56)^1^

Eye Symptom	Group	Baseline (SD)	At 12 Wks (SD)	Difference (SD)	P Value 1^2^	P Value 2^3^
Blurred Vision	A	2.1 (1.2)	0.5 (0.6)	-1.6 (1.2)	0.0095	0.2712
	B	1.8 (1.2)	0.5 (0.8)	-1.3 (1.2)	0.1215	-
	C	1.7 (1.2)	0.7 (0.8)	-1.5 (1.2)	-	-
Dryness	A	3.0 (1.1)	0.9 (0.9)	-2.2 (1.4)	< 0.0001	0.0281
	B	3.0 (1.1)	1.3 (0.7)	-1.6 (1.3)	0.0082	-
	C	2.5 (1.0)	1.5 (0.8)	-1.0 (1.3)	-	-
Itching	A	2.3 (1.2)	0.5 (0.7)	-1.8 (1.5)	0.0137	0.3903
	B	2.6 (1.0)	0.9 (1.0)	-1.7 (1.1)	0.0310	-
	C	2.1 (1.1)	0.8 (0.7)	-1.3 (1.0)	-	-
Photosensitivity	A	2.5 (1.3)	0.6 (0.6)	-1.9 (1.4)	0.0012	0.0179
	B	2.4 (1.1)	1.0 (1.0)	-1.3 (1.2)	0.2295	-
	C	2.0 (1.2)	0.9 (0.7)	-1.1 (1.1)	-	-
Pain/Soreness	A	2.2 (1.2)	0.3 (0.5)	-1.9 (1.1)	0.0351	0.1171
	B	2.2 (1.2)	0.6 (0.9)	-1.5 (1.2)	0.5748	-
	C	2.2 (1.2)	0.6 (0.6)	-1.4 (1.1)	-	-
Sandy/Gritty	A	2.6 (1.1)	0.7 (0.6)	-2.0 (1.4)	0.0014	0.0086
	B	2.4 (1.2)	1.1 (1.0)	-1.3 (1.2)	0.3702	-
	C	2.3 (1.2)	1.2 (0.7)	-1.1 (1.3)	-	-
Stinging/Burning	A	2.6 (1.2)	0.6 (0.7)	-2.0 (1.3)	0.0010	0.0493
	B	2.4 (1.4)	1.0 (1.0)	-1.5 (1.4)	0.1485	-
	C	2.5 (1.3)	1.1 (0.7)	-1.2 (1.2)	-	-

^[1]^ Each eye scored separately

^[2]^ Wilcoxon’s rank sum test: groups A and B vs. group C

^[3]^ Wilcoxon’s rank sum test: group A vs. group B

### Best corrected visual acuity

BCVA was monitored in all patients at each visit using ETDRS charts in order to detect any effect the test product or effective treatment of DED may have on visual acuity. The results were small and inconsistent, with none of the treatment groups demonstrating a clear or consistent treatment effect on BCVA, and no appreciable divergence between groups.

### Clinician’s global evaluation of treatment

Investigator’s global impressions on the effectiveness of treatment in each eye was captured through a questionnaire completed by the investigating ophthalmologist. The results are shown in Table 4. Although a trend favouring test group A is visible, this did not reach a level of definitive statistical confidence by the end of 12 weeks of treatment.

**Table 4:** Clinician’s Global Evaluation of Treatment: End of Week 12†

	ITT population			PP population		
Treatment group	A	B	C	A	B	C
Number of eyes graded	96	98	96	60	64	56
Completely cleared	14 (14.6%)	2 (2.0%)	4 (4.2%)	14 (23.3%)	2 (3.1%)	4 (7.1%)
~ 90% improvement	21 (21.9%)	23 (23.5%)	21 (21.9%)	19 (31.7%)	23 (35.9%)	17 (30.3%)
~ 75% improvement	11 (11.5%)	18 (18.4%)	29 (30.2%)	9 (15.0%)	16 (25.0%)	21 (37.5%)
~ 50% improvement	13 (13.5%)	17 (17.3%)	11 (11.5%)	13 (21.7%)	17 (26.6%)	11 (19.6%)
~ 25% improvement	3 (3.1%)	3 (3.1%)	3 (3.1%)	3 (5.0%)	3 (4.7%)	3 (5.4%)
Condition unchanged	2 (2.1%)	3 (3.1%)	0 (0.0%)	2 (3.3%)	3 (4.7%)	0 (0.0%)
Condition worsened	0 (0.0%)	0 (0.0%)	0 (0.0%)	0 (0.0%)	0 (0.0%)	-
P-Value 1	0.1576	0.3075	-	0.1760	0.4363	-
P-Value 2	0.0278	-	-	0.0364	-	-

† Last observation was carried forward in case of missing data in the ITT population.

1 Wilcoxon’s rank sum test: test groups vs. control

2 Wilcoxon’s rank sum test: test group A vs. test group B

### Use of tear substitutes

The protocol of this study permitted the use of tear substitutes. An adequate supply of Refresh Tears™ (carboxymethylcellulose sodium 0.5% w/v, Allergan India Pvt. Ltd) was provided to patients at each visit and the empty vials retrieved at follow-up visits. The mean count of vials retrieved at the 1^st^ follow-up visit 4 weeks into the trial ranged from 4.46 to 5.03 across treatment groups with the median at 5 vials for each group. Consumption of tear substitutes remained stable in all groups through the course of the trial, showing neither any appreciable change within groups at subsequent visits nor divergence between treatment groups.

### Compliance

Treatment compliance was calculated on the basis of data captured on patient diaries. The number of doses administered, as a proportion of the number of doses prescribed, is expressed as a percentage. Some patients failed to maintain diaries and had to be eliminated from the analysis. In general, the number of patients with missing data was comparable across treatment groups at the various time-points for analysis in the ITT and PP populations. For analysis of compliance at the end of 12 weeks in the PP population, data was missing for 2, 3, and 0 patients in treatment groups A, B, and C, respectively. Excluding these patients, compliance was found to be 98.3%, 99.0%, and 95.9% in the 3 treatment arms, respectively.

### Efficacy confidence intervals

**Fig. 1:**
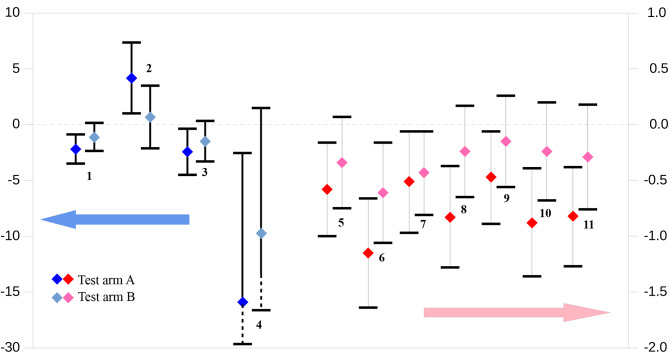
Difference in mean scores versus control with 95% confidence intervals^†^ ^†^ PP population at 12 weeks. 1 = CFSS; 2 = STT; 3 = CLGS; 4 = OSDI; 5 = Blurring; 6 = Dry eyes; 7 = Itching; 8 = Photophobia; 9 = Painful/sore eyes; 10 = Sandy/gritty eyes; 11 = Stinging/burning.

Figure 1 shows 95% confidence intervals for difference between mean change in efficacy outcome scores of test and control arms at 12 weeks (PP population). Darker shades of blue and red represent the difference between means of the higher dose test arm and control arm (treatment groups A and C), and the lighter shades represent the difference between means of the lower dose test arm and control arm (treatment groups B and C). The scale on the left of the figure maps the CIs for difference in means of objective endpoints and patient-reported OSDI (blue dots), while the scale on the right of the figure is for difference in means of individual symptom scores (red dots). As is evident from the figure, 95% CIs of difference in means between the higher dose test arm and control excluded 0 for all the mapped outcome scores. For means between the lower dose test arm and control this was only true for the feeling of dryness and itching in the eyes.

### Safety and toleration

A total of 39 instances of adverse events were reported in 24 patients during the course of the study. None of these met criteria for seriousness. Other than 2 events in 2 patients in the control group that were judged by the investigators to be of moderate severity, all events were mild. None of the events required interruption or discontinuation of study medication.

Both events that were judged to be of moderate severity related to pyrexia presumed to be of infections origin not requiring hospitalization. Both events occurred in the control group several weeks after start of study medication, and resolved within 48 h with supportive therapy. Relationship to study drugs was judged by the investigators to be unlikely for both events and the study medication was not withdrawn in either case.

Of the 39 events reported during the course of the study, 14 related to eye discomfort coded as irritation, discharge, pruritus, excessive lacrimation, or hyperaemia. As shown in Table 5, the number of patients reporting these events was too small for meaningful statistical analysis. Other events reported during the trial were of the nature of common extra-ocular or systemic ailments, and included instances of headache (4), gastrointestinal symptoms (4), nasopharyngeal symptoms (3) and pyrexia (3), joint and back pain (3), cough and wheezing (2), pruritus (1), insomnia (1), ligament sprain (1), hypocalcemia (1), hypertension (1) and anaemia (1). The distribution of these events across treatment groups is shown in Table 5.

**Table 5:** Safety and Toleration: Number of adverse events and patients reporting events

Treatment Arm	A	B	C
Safety population size (N)	51	53	51
Total number of events	12	12	15
Patients reporting events (%)	7 (13.7)	7 (13.2)	10 (19.6)
Events causing eye discomfort	5	6	3
Patients reporting events causing eye discomfort (%)	3 (5.9)	2 (3.8)	2 (3.9)
Extra-ocular/systemic events	7	6	12
Patients reporting extra-ocular/systemic events (%)	6 (11.8)	4 (7.5)	10 (19.6)

None of the inter-group differences in the number of ocular and extra-ocular adverse events or the number of patients reporting such events was found to be statistically significant at an α value of 0.05 using a 2 × 2 chi-square test with Yates correction.

## Discussion 

The advent of topical cyclosporine into the therapeutic armamentarium available for patients with dry eye disease almost 2 decades ago was a significant advance in the treatment of keratoconjunctivitis sicca. Although approved specifically to increase tear production believed to be suppressed due to ocular inflammation associated with keratoconjunctivitis sicca, the broad effects of its antinflammatory action on the pathophysiology of the disease is now well recognized [13]. Indeed, recognition of the central role that inflammation plays in the pathophysiology of dry eye disease has made control of inflammation mandatory in order to improve symptomatology [14]. Products currently available for the control of ocular inflammation in DED do have their shortcomings, however. Corticosteroids are not recommended for long-term use due to the risk of a variety of side effects [15], and use of eye drops containing fatty acids (omega−3, eicosapentaenoic and docosahexaenoic acids) is considered supplementary and plagued by uncertainty about efficacy [16]. Cyclosporine and lifitegrast are the only non-steroidal immunomodulating antiinflammatory drugs available in ophthalmic formulation that have received regulatory approval for use in dry eye disease. Both were approved on the basis of placebo-controlled trials that proved efficacy but also documented a considerable burden of issues with local tolerability [5, 17]. Data from these trials leaves substantial scope for improvements in efficacy and tolerability of these drugs through the application of formulation technology that would enhance penetration of the drug substance into target tissues while reducing surface irritation.

We compared two dose levels of a proprietary formulation of MNP cyclosporine with Restasis™, a commonly used and widely available ophthalmic preparation of cyclosporine. The study enrolled patients between May 2019 and March 2020 and patient follow-up visits extended into September 2020. Hence the follow-up visits of many patients were disrupted by pandemic-related restrictions, leading to a greater number of patient drop-outs than previously anticipated. Nevertheless, the loss of patient numbers was evenly distributed across treatment arms. Moreover, the final sample size proved to be sufficient to detect a strong efficacy signal for all the treatment arms as well as to detect divergence among treatment arms.

Paired t tests of follow-up data versus baseline values showed a strong efficacy signal (p ≤ 0.0005) for all treatment arms for all objective (CFS, STT, CLGS) and subjective measures of disease activity (OSDI, DESS), attesting to the sensitivity of the endpoints to therapeutic change.

The primary efficacy endpoint, CFS, showed a superior outcome with the higher dose regimen of MNP cyclosporine (one drop of 0.05% twice daily) compared to Restasis™ at the same dose in the PP population at 12 weeks (p = 0.0026). The conclusion from this outcome was supported by outcomes documented for both objective and patient-reported outcomes in the secondary efficacy endpoints (STT p = 0.01; CLGS p = 0.02; OSDI p = 0.02) assessed for the same population at the same time point. All individual symptom scores also pointed to the same conclusion. The superiority signal was stronger for all objective endpoints on ANCOVA using baseline scores as the covariate. Thus, this study endorsed the hypothesis that manipulation of formulation characteristics can influence treatment outcomes and enhance the therapeutic value of topically administered cyclosporine in the management of dry eye disease.

Superiority of efficacy of MNP cyclosporine over Restasis™ was not sustained at the lower dose of the test product (1 drop twice daily for 4 weeks and once daily thereafter). A separation between outcomes in the lower dose MNP cyclosporine and control arms was not evident at 12 weeks for any of the objective endpoints or for the patient-reported OSDI, and for only 2 of the 7 individual DES scores. Consequently, the higher dose of MNP cyclosporine was numerically more effective than the lower dose of the test product for all the endpoints, and the separation between the doses was statistically significant for STT and 4 of 7 individual symptom scores. Thus, this dose-finding study provides unambiguous data for the choice of dose in patients with moderate to severe keratoconjunctivitis sicca.

We found no effect of treatment on BCVA or on use of tear substitutes. BCVA was close to 0.3 logMAR at baseline for each of the 3 treatment arms. Lack of appreciable improvement in visual acuity despite substantial improvements in the signs and symptoms of DED suggests that the deviation from normal vision seen in this patient population at baseline may not have been attributable to DED. The lack of reduction in use of tear substitutes is more difficult to explain. A 2019 review of studies with cyclosporine in DED [18] in the Cochrane Database of Systematic Reviews included 3 studies that reported on the effect of cyclosporine treatment on use of artificial tears. Two of these found no statistically significant difference in the use of artificial tears between test and control groups at 6 months, while 1 reported a statistically significant outcome in favor of the intervention. The authors found certainty of evidence to be low and the studies were downgraded for inconsistency and risk of bias.

This study did not reveal any issues with safety or tolerability of the test product. All study treatments seemed to have been reasonably well tolerated. We could not detect any differences in toleration of the test product in comparison to the control group. This study failed to substantiate the hypothesis that differences in formulation characteristics would confer a superior local toleration profile on the test product. The sample size of this study may have been insufficient to detect differences in safety and tolerability of the study drugs.

A number of limitations of this study may need to be kept in mind while interpreting the results. Foremost among these is perhaps the open-label design that may have permitted conscious and unconscious biases to affect subject selection and assessment of endpoints. Although the primary efficacy endpoint and two of the secondary endpoints were of an objective nature requiring precise or semi-precise readouts using instrumental techniques, the influence of bias cannot be entirely ruled out. Nevertheless, it would be reassuring to note that the main conclusions to be drawn from this study do not depend on those endpoints that may be expected to be more vulnerable to human bias, namely patient-reported outcomes and the CGET, although the patient-reported outcomes strongly support the results of objective endpoints. Indeed, it is interesting to note that the endpoint that could be considered the most susceptible to bias, namely CGET, did not return a categorical outcome.

Another limitation of the study is the large number of patients that had to be excluded from the PP analysis, and the curtailment of efficacy analysis to the 12-week time-point, resulting from disruptions consequential to civic restrictions imposed due to the coronavirus pandemic. It is possible that a longer duration of follow-up for a larger set of patients could have narrowed the divergence between the two dose-levels of the test product and between the test and control groups by allowing more time for the slower-acting formulations to catch up with the lead group. On the other hand, it can be argued that the differences between treatment groups seen at 12 weeks in the present analysis may have been consolidated over a longer follow-up period. Further studies to test these hypotheses may be in order.

It has been customary in ophthalmology, and other disciplines where diseases affecting paired units are the subject of analysis, to include only one unit (usually the more severely affected limb or member) from each individual in efficacy assessments. This is to ensure that each value included in the analysis is an independent data-point unrelated to the movement of other data-points. This is a necessary prerequisite to the proper use of common statistical tests of inference that are rendered invalid if this rule is overlooked, since the presence of interdependent values inappropriately inflates the true size of the sample [19]. On the other hand, interdependency of outcomes between the two eyes is rarely absolute, and data is inevitably lost when only one eye is considered for analysis [20]. In the present study both eyes were included for calculation of sample size and analysis of endpoints. Thus the true size of differences between treatment groups may have been inflated by a factor of up to 2. To check whether this would make a difference to the primary conclusions to be drawn from the study, we ran the statistical test of inference (Student’s t test for independent samples) on the primary endpoint (comparing mean change in CFS score in test and control groups) at half the sample size, using the same standard deviations. The p value for difference between test group A and the control group in mean change in CFSS from baseline in this analysis was 0.0322, indicating that the primary conclusions of the study would still hold if only one eye or an average of two eyes was used for the analysis.

## Conclusions

MNP Cyclosporine is a novel proprietary micellar nano-particulate formulation of cyclosporine designed for topical ophthalmic use. This study provides documentation of the efficacy and safety of MNP Cyclosporine in the treatment of patients with moderate to severe keratoconjunctivitis sicca. The results indicate that the dose of 1 drop of a 0.05% w/v ophthalmic emulsion administered topically twice daily yields better outcomes at 12 weeks than the lower dose tested in the study, and is more efficacious than an equivalent dose of Restasis™, the active control used in the study.

## Data Availability

All data necessary to interpret, replicate and build upon the findings of this study are summarized in this published article. The datasets analysed and the full protocol are available from the corresponding author on reasonable request.

## Abbreviations

AE: adverse event
ANCOVA: analysis of covariance
BCVA: best corrected visual acuity
CFS/CFSS: corneal fluorescein staining score
CGET: clinician’s global evaluation of treatment
CI: confidence interval
CLGS: conjunctival lissamine green staining
CsA: cyclosporine A
CTCAE: common terminology criteria for adverse events
DED: dry eye disease
DESS: dry eye symptom score
ETDRS: early treatment diabetic retinopathy study
ICH: international conference on harmonization
IEC: institutional ethics committee
ITT: intension to treat
KCS: keratoconjunctivitis sicca
MAR: minimum angle of resolution
MedDRA: medical dictionary for regulatory activities
MNP: micellar nano-particulate
NCI: national cancer institute
NEI: national eye institute
OSDI: ocular surface disease index
PP: per-protocol
SOC: system organ class
STT: Schirmer tear test
USFDA: United States Food and Drug Administration
WHO-UMC: World Health Organisation Uppsala Monitoring Centre
